# Hematite (α‐Fe_2_O_3_) Nanoparticles Conjugated with Thiosemicarbazones: Hydrothermal‐Assisted Synthesis, Characterization, Antibacterial, Antioxidant, and Photocatalytic Activities

**DOI:** 10.1002/cbdv.71822

**Published:** 2026-10-15

**Authors:** Ekhlakh Veg, Srishti Dwivedi, Smita Rai, Swati Sharma, Nashra Fatima, Seema Joshi, Abdul Rahman Khan, Tahmeena Khan

**Affiliations:** ^1^ Department of Chemistry Integral University Lucknow Uttar Pradesh India; ^2^ Department of Chemistry Isabella Thoburn College Lucknow Uttar Pradesh India; ^3^ Department of Chemistry Babasaheb Bhimrao Ambedkar University Lucknow Uttar Pradesh India; ^4^ Department of Biosciences Integral University Lucknow Uttar Pradesh India

**Keywords:** biological activity, environmental remediation, nanoconjugates, photocatalysis, α‐Fe_2_O_3_ NPs

## Abstract

In the present study, pre‐synthesized hematite (α‐Fe_2_O_3_) nanoparticles (NPs) were functionalized with three thiosemicarbazone ligands (TSCs), namely salicylaldehyde thiosemicarbazone (STSC), vanillin thiosemicarbazone (VTSC), and 3‐acetylpyridine thiosemicarbazone (3‐APTSC), through a hydrothermal conjugation approach. The resulting α‐Fe_2_O_3_/TSC nanoconjugates were characterized using UV–visible (UV‐Vis), Fourier Transform Infrared (FT‐IR), X‐ray diffraction (XRD), and scanning electron microscopy coupled with energy‐dispersive x‐ray spectroscopy (SEM–EDX) analytical techniques. XRD analysis confirmed the crystalline nature of the nanoconjugates, with average crystallite sizes of 15.27 nm, 12.47 nm, and 11.15 nm for α‐Fe_2_O_3_/STSC, α‐Fe_2_O_3_/VTSC, and α‐Fe_2_O_3_/3‐APTSC, respectively. UV–vis analysis revealed absorption maxima at 297, 324, and 229 nm, with corresponding optical band‐gap energies estimated at 2.66, 3.38, and 2.52 eV for the nanoconjugates. FT‐IR and EDX analyses confirmed successful surface functionalization of α‐Fe_2_O_3_ NPs with the respective TSCs. The nanoconjugates exhibited concentration‐dependent antibacterial activity against *Bacillus subtilis*, *Staphylococcus aureus*, *Escherichia coli*, and *Salmonella abony*, with α‐Fe_2_O_3_/3‐APTSC showing the highest antibacterial efficacy. Antioxidant evaluation using the DPPH assay demonstrated 63.97%, 42.96%, and 74.47% radical‐scavenging activities for α‐Fe_2_O_3_/STSC, α‐Fe_2_O_3_/VTSC, and α‐Fe_2_O_3_/3‐APTSC, respectively, whereas the same conjugates exhibited 65%, 63%, and 68% degradation of malachite green (MG) dye under visible‐light irradiation in 90 min. Overall, α‐Fe_2_O_3_/3‐APTSC exhibited the most promising overall biological and photocatalytic performance. These findings demonstrate that thiosemicarbazone functionalization provides an effective strategy for enhancing the multifunctional properties of hematite NPs for potential environmental and biomedical applications.

## Graphical abstract

The study describes the functionalization of α‐Fe_2_O_3_ nanoparticles with three different thiosemicarbazone ligands through a hydrothermal route. The nanoconjugates were tested for their antibacterial and antioxidant activities, and their photocatalytic potential was tested against malachite green dye. Overall, α‐Fe_2_O_3_/3‐APTSC exhibited the most promising overall biological and photocatalytic activity.

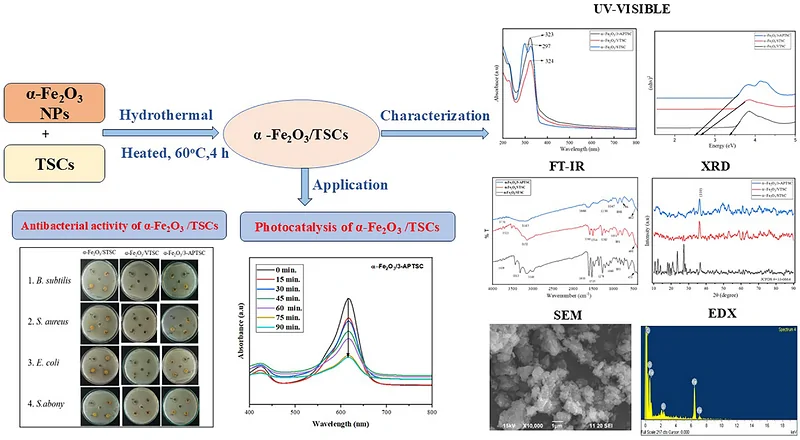

## 1 Introduction

Iron oxides occur in several polymorphic forms, among which α‐Fe_2_O_3_ (hematite), β‐Fe_2_O_3_, γ‐Fe_2_O_3_ (maghemite), and Fe_3_O_4_ (magnetite) are well‐known crystalline phases [1]. α‐Fe_2_O_3_, the most stable iron oxide phase, exhibits chemical stability, semiconducting behavior with a narrow band gap (∼2.0–2.2 eV), and photocatalytic activity under visible light [2]. Unfunctionalized iron oxides tend to degrade, leach, and agglomerate due to magnetic dipole–dipole interactions, resulting in poor stability, dispersibility, and limited biocompatibility. Iron oxide NPs, particularly α‐Fe_2_O_3_, have attracted considerable attention due to their diverse applications in environmental, biomedical, and catalytic fields. In environmental remediation, α‐Fe_2_O_3_‐based nanomaterials (NMs) have been extensively investigated as photocatalysts and adsorbents for the degradation and removal of organic pollutants, dyes, pesticides, and pharmaceutical contaminants from wastewater owing to their visible‐light responsiveness, chemical stability, and low cost. In the biomedical field, iron oxide nanoparticles have been explored as antimicrobial, antioxidant, drug‐delivery, bioimaging, and therapeutic agents because of their biocompatibility and ease of surface modification. Furthermore, α‐Fe_2_O_3_ nanostructures have found applications in sensors, energy‐storage devices, photoelectrochemical water splitting, and heterogeneous catalysis, where their semiconducting nature and tunable surface properties play a crucial role. These versatile applications have stimulated growing interest in developing surface‐functionalized α‐Fe_2_O_3_ nanomaterials with enhanced stability and multifunctional performance. In particular, Fe_3_O_4_ is prone to oxidation and can lose its magnetic properties in air. To improve their colloidal stability and usability in biological systems, surface functionalization is essential. This can be achieved using natural biopolymers such as citric acid, dextran, chitosan, alginate, gelatin, and lignin, as well as synthetic polymers like polyvinylpyrrolidone (PVP), polyvinyl alcohol (PVA), and polyethene glycol (PEG), which enhance particle stability and biocompatibility [3]. Their ability to coordinate with nitrogen‐ and oxygen‐containing ligands makes them suitable for functionalization with Schiff bases, like TSCs, and related organic moieties for biomedical and environmental applications [4]. TSCs and their metal complexes have received considerable research attention because of their diverse biological properties, including antimicrobial, anticancer, and antioxidant activities. These activities can be further enhanced due to structural variations like conjugation with NPs or changing substituents on TSC moieties. Benzaldehyde‐derived TSCs are particularly significant as their biological performance is strongly influenced by the nature and position of substituents on the phenyl ring, with electron‐donating and hydroxyl groups enhancing activity [5, 6]. There are various methods available for the synthesis of iron oxide–TSC conjugates, including precipitation, sol–gel, and ultrasonication techniques. However, the hydrothermal method is an efficient approach for synthesizing iron oxide NMs, enabling precise control over particle size, morphology, crystallinity, and surface functionalization under mild, aqueous conditions. This method also facilitates the homogeneous incorporation of organic ligands, yielding stable nanocomposites with tailored properties [7]. Hydrothermal synthesis is a widely employed method for preparing nanomaterials due to its simplicity, cost‐effectiveness, and environmentally friendly nature, as water serves as the solvent. This technique involves the formation of nanostructures over a broad temperature range, from ambient to high temperatures. By adjusting the reaction conditions, including low‐ or high‐pressure environments, the morphology, size, and crystallinity of the resulting nanomaterials can be precisely controlled [8]. Hydrothermally synthesized GdBO_3_–Fe_3_O_4_ nanocomposite functionalized with citric acid, fluorescein‐doped silica, and folic acid exhibited favorable magnetic properties, high boron and gadolinium content, and efficient cellular uptake. These multifunctional nanocomposites show potential for targeted drug delivery, magnetic resonance imaging (MRI), and boron/gadolinium neutron capture therapy (BNCT/GdNCT) [9]. The antibacterial potential of Fe_3_O_4_@Glu–TSC magnetic NPs was evaluated against methicillin‐resistant *Staphylococcus aureus* (*S. aureus*). Functionalization with glutamic acid and conjugation with thiosemicarbazide significantly enhanced the antibacterial effect compared to bare Fe_3_O_4_ NPs. The Minimum Inhibitory Concentration (MIC) values of Fe_3_O_4_ @Glu–TSC MNPs were reduced by twofold, demonstrating higher potency in inhibiting bacterial growth. These results indicate that the combination of the Fe_3_O_4_ core with bioactive glutamic acid and thiosemicarbazide ligands created a synergistic effect that effectively suppresses MRSA proliferation, highlighting the potential of Fe_3_O_4_ @Glu–TSC MNPs as an efficient antibacterial agent [10]. Fe_3_O_4_@Glu/BTSC exhibited stronger cytotoxic effects against A549 lung cancer cells than either BTSC or Fe_3_O_4_ NPs alone, with an IC_50_ of 166.77 µg/mL, while showing lower toxicity toward healthy cells (CC_50_ = 189.15 µg/mL), resulting in a selectivity index of 1.13. Treatment with Fe_3_O_4_ @Glu/BTSC induced a higher apoptosis rate (46.33%) compared to BTSC (28.27%) and Fe_3_O_4_ (26.02%), and effectively arrested cell cycle progression in the Sub‐G1 and S phases. Additionally, expression of the tumor‐suppressor microRNA let‐7c was significantly upregulated in treated cells, increasing 6.9‐fold with Fe_3_O_4_@Glu/BTSC, compared to 2.2‐fold with BTSC and 1.6‐fold with Fe_3_O_4_. These findings indicate that Fe_3_O_4_ @Glu/BTSC enhanced apoptosis, modulated the cell cycle, and promoted let‐7c expression, demonstrating potent anti‐proliferative activity against lung cancer cells [11]. In another study, glutamic acid‐functionalized ZnO NPs conjugated with TSC (ZnO@Glu‐TSC) were successfully synthesized and characterized using FT‐IR, XRD, EDS, and zeta potential analysis. The conjugated NPs exhibited enhanced antibacterial activity against *Pseudomonas aeruginosa*, particularly when combined with thymol, showing MIC of 3.12–25.5 µg/mL. Furthermore, the combined treatment inhibited biofilm formation by up to 95%, demonstrating the potential of thiosemicarbazone‐functionalized metal oxide nanomaterials as effective antibiofilm and antibacterial agents [12]. Similarly, in another study, NiO@Glu/TSC NPs exhibited higher toxicity toward AGS gastric cancer cells (IC_50_ = 220 µg/mL) compared to normal fibroblast cells (IC_50_ = 390 µg/mL). Apoptosis analysis showed that treated AGS cells underwent late apoptosis with nuclear fragmentation and clumping, confirming the effective anti‐proliferative activity of NiO@Glu/TSC NPs [13]. Magnetic Fe_3_O_4_ NPs were functionalized and conjugated with BTSC to form Fe_3_O_4_@SiO_2_–APTES–BTSC, which demonstrated a high adsorption capacity for Pd (II) ions. Additionally, BTSC was successfully attached to SiO_2_ at the dicarbonyl backbone and to α‐Fe_2_O_3_ at the diamine tail, yielding SiO_2_@APTES–BTSC and Fe_2_O_3_–dopamine–BTSC, respectively. The SiO_2_@APTES–BTSC–Pd complex served as an efficient heterogeneous catalyst in the Suzuki–Miyaura cross‐coupling reaction, with XPS analysis confirming that Pd existed predominantly in the elemental state. Furthermore, metal sulfide NPs including Cu_7_._2_S_4_, Pd_16_S_7_, and ZnS were successfully prepared through microwave‐assisted decomposition of the pentadentate BTSC metal complexes [14]. In another study, Co_3_O_4_@Glu/TSC nanocomposites showed dose‐dependent cytotoxicity against AGS gastric cancer cells, with an IC_50_ value of 107.5 µg/mL. Apoptosis evaluation through Annexin V/PI staining, caspase‐3 activation, and Hoechst 33258 staining showed that treated AGS cells underwent significant apoptosis, including nuclear fragmentation and chromatin condensation. Co_3_O_4_@Glu/TSC exhibited strong cytotoxic and anti‐cancer activity against AGS cells, suggesting potential application in targeted drug delivery systems [15]. In another study, α‐Fe_2_O_3_/graphene oxide (α‐Fe_2_O_3_/GO) nanocomposite was fabricated via a facile hydrothermal method and evaluated for the removal of ionic dyes, Congo red (CR) and Methyl violet (MV), from aqueous solutions. The characterization confirmed successful integration of α‐Fe_2_O_3_ with GO, providing a thin morphology and abundant oxygen‐containing groups. The α‐Fe_2_O_3_/GO nanocomposite exhibited superior adsorption performance compared to α‐Fe_2_O_3_ or GO alone, achieving rapid equilibrium in 8 min for CR (95.78%, 239.45 mg·g^−^^1^) and 5 min for MV (99.91%, 1998.14 mg·g^−^^1^). Adsorption was spontaneous and endothermic, primarily driven by hydrogen bonding and π–π interactions, with kinetics controlled by surface reactions. The synergistic effect between α‐Fe_2_O_3_ and GO, combined with simple fabrication and high cycling stability, highlights α‐Fe_2_O_3_/GO as a promising adsorbent for efficient organic dye removal in wastewater treatment [16]. Although several studies have reported the synthesis of thiosemicarbazone‐functionalized metal oxide NPs, investigations involving hydrothermally conjugated α‐Fe_2_O_3_/TSCs remain limited. In particular, comparative studies evaluating the influence of structurally different TSC ligands on the antibacterial, antioxidant, and photocatalytic performance of α‐Fe_2_O_3_ NPs are limited. Therefore, the development of multifunctional α‐Fe_2_O_3_/TSC nanoconjugates and the assessment of their structure–activity relationships remain important areas of investigation. This work investigates the biological and photocatalytic properties of pre‐synthesized α‐Fe_2_O_3_ NPs [17] upon conjugation with the three TSCs [18]. The NPs were fabricated using *Livistona chinensis* leaf extract, as described earlier [17].

## 2 Materials and Methods

The materials and methods used for the synthesis of TSC ligands and α‐Fe_2_O_3_ NPs are reported previously [17, 18]. Hanker hydrothermal reactor with 50 mL capacity was used for the synthesis of nanoconjugates. All the chemicals required for photocatalytic and biological activity are also described previously [18].

## 3 Experimental Section

### 3.1 Synthesis of TSCs

The ligands were synthesized by the condensation of equimolar amounts (0.01 M) of the respective carbonyl compounds (salicylaldehyde, vanillin, and 3‐acetylpyridine) with thiosemicarbazide in absolute ethanol [18]. A few drops of glacial acetic acid were added as a catalyst, and the reaction mixtures were refluxed at 70°C–80°C for 4 h. The resulting products were cooled, filtered, washed with ethanol, and dried under ambient conditions as given in Figure 1. Compared with previously reported procedures, the modified reflux conditions provided improved yields. The synthesized ligands were subsequently characterized as reported earlier and used for the preparation of nanoconjugate [18].

**FIGURE 1:**
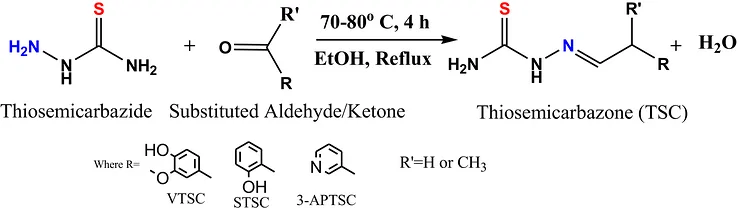
General scheme for synthesis of Thiosemicarbazone ligands.

### 3.2 Synthesis of α‐Fe_2_O_3_ NPs

α‐Fe_2_O_3_ NPs were synthesized via a green‐mediated coprecipitation route using *L. chinensis* leaf extract [17] using Fe(NO_3_)_3_·9H_2_O as the precursor in basic medium (pH = 8–10). The formed NPs were centrifuged, washed with distilled water and ethanol, dried at 80°C, and subsequently calcined at 500°C–600°C for 3 h to obtain crystalline α‐Fe_2_O_3_ NPs.

### 3.3 Synthesis of α‐Fe_2_O_3_/TSC Nanoconjugates

α‐Fe_2_O_3_/TSC nanoconjugates were prepared by mixing α‐Fe_2_O_3_ NPs (200 mg) and the respective TSC ligands (600 mg) in a 1:3 molar ratio. The mixtures were stirred thoroughly to achieve uniform dispersion and transferred into a 50 mL hydrothermal reactor, followed by heating at 60°C for 4 h to promote surface coordination, capping and nanocomposite formation. The resulting products were separated by centrifugation, washed with distilled water and ethanol to remove excess or unbound ligand molecules, and oven‐dried at 90°C to obtain the α‐Fe_2_O_3_/TSC conjugates as fine dry powders. The schematic diagram of the formation of α‐Fe_2_O_3_/TSC conjugates is given in Figure 2, and the proposed mode of conjugation is given in Figure 3.

**FIGURE 2:**
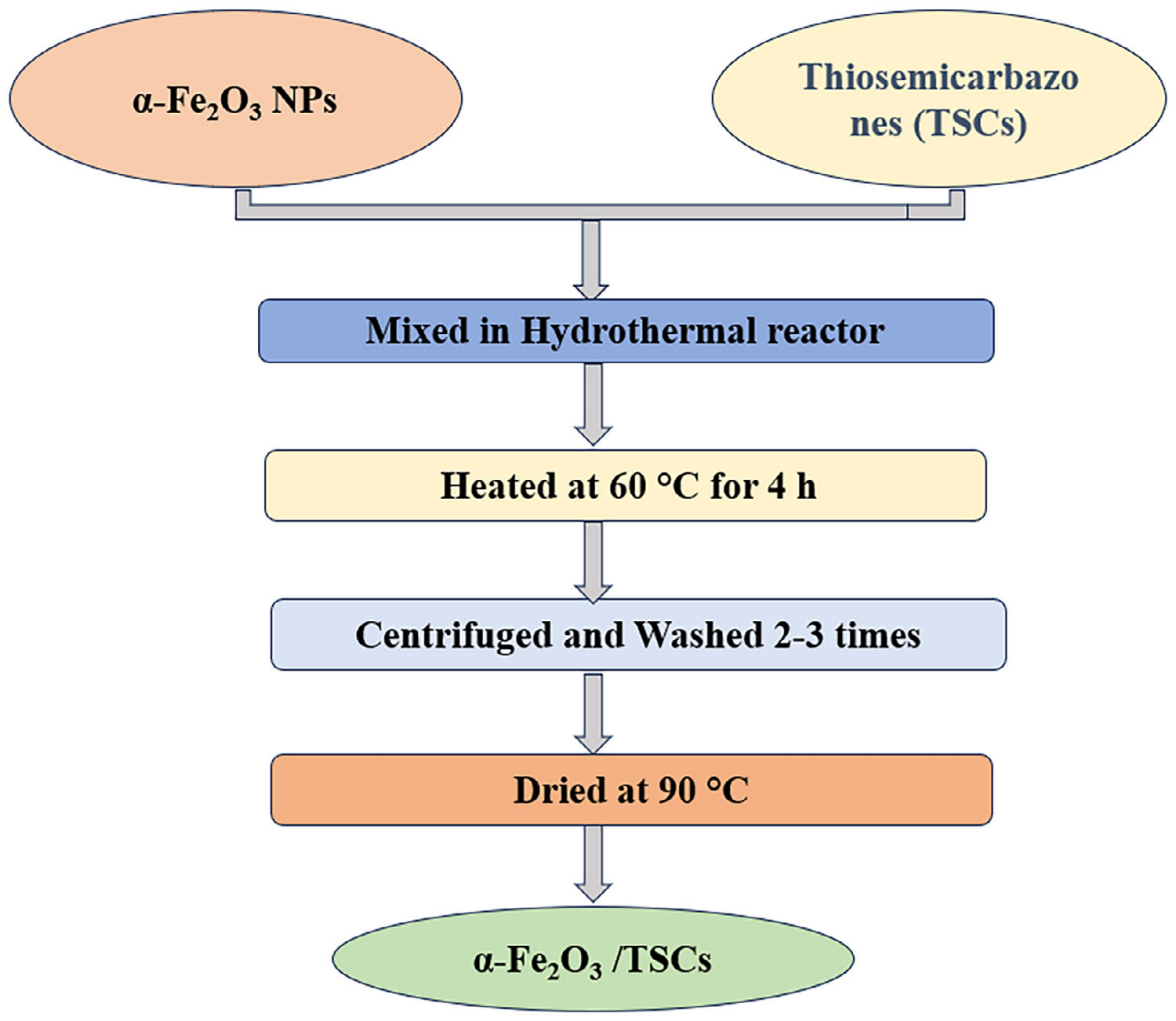
Schematic representation of the synthesis of nanoconjugates.

**FIGURE 3:**
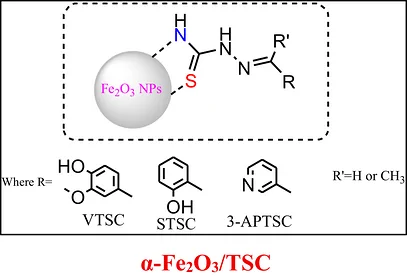
Illustration of the formation of α‐Fe_2_O_3_/TSC nanoconjugates.

### 3.4 Antibacterial Activity Evaluation

The antibacterial activity was evaluated using the agar well diffusion method and microdilution [18].

### 3.5 Assessment of Antioxidant Activity Using DPPH RSA

Antioxidant activity was evaluated using the previously described methodology [18]. The scavenging percentage was obtained using Equation (1):

(1) $$\%\;Antioxidant\;activity=\left[\left(A_{c}-A_{s}\right)÷A_{c}\right]\times 100$$

where, *A*_c_ = absorbance of control and *A*_s_ = absorbance of test sample.

### 3.6 Statistical Analysis

All experiments were conducted in triplicate, and the results are expressed as the mean ± standard error of the mean (SEM) from three independent experiments. Error bars represent the SEM. Statistical significance was evaluated using two‐way analysis of variance (ANOVA) followed by an appropriate multiple comparison test. Differences with a *p* value < 0.05 were considered statistically significant.

### 3.7 Assessment of Photocatalytic Dye Degradation

The photocatalytic activity of the synthesized NPs was evaluated by monitoring the degradation of MG dye under visible light irradiation. Briefly, 25 mg of the photocatalyst was dispersed in 50 mL of a 10 ppm MG solution. Photocatalytic experiments were conducted under visible light irradiation using a Philips white LED bulb (9 W, 220–240 V). The light source was positioned 30 cm from the reaction vessel inside a dark wooden enclosure to minimize interference from ambient light and ensure uniform irradiation conditions. Before light exposure, the suspension was magnetically stirred in the dark for 30 min to achieve adsorption–desorption equilibrium between the catalyst surface and dye molecules. The absorbance measured immediately after illumination was considered the initial concentration (*C*_0_, *t* = 0 min). During photocatalysis, 3 mL aliquots were collected at 15 minutes' intervals, and the absorbance of the degraded dye solution was recorded over the wavelength range of 200–800 nm [18]. The degradation efficiency was calculated using Equation (2):

(2) $$\%Degradation=\left(A_{o}-A_{t}\right)/A_{o}$$

where *A*_o_ and *A_t_* represent the absorbance of the dye at the initial time and at time ‘*t*’, respectively. The photocatalytic degradation kinetics were analyzed using the pseudo‐first‐order kinetic model by plotting ln(*C*_o_/*C_t_*) versus irradiation time, as expressed in Equation (3):

(3) $$ln\left(C_{0}/C_{t}\right)=kt$$

where *C*_0_ and *C_t_* denote the dye concentrations at the initial time and at time ‘*t*’, respectively, and *k* is the apparent rate constant, which was determined from the slope of the linear plot.

## 4 Results and Discussion

### 4.1 UV–Visible Analysis

The UV–Vis spectra of the synthesized α‐Fe_2_O_3_/3‐APTSC, α‐Fe_2_O_3_/VTSC, and α‐Fe_2_O_3_/STSC showed absorption maxima at 323 nm, 324 nm, and 297 nm, respectively (Figure 4a), which are consistent with ligand‐to‐metal charge transfer (LMCT) and π → π* transitions of coordinated thiosemicarbazone moieties. Experimental band gaps obtained from the Tauc's plots were 2.52 eV (α‐Fe_2_O_3_/3‐APTSC), 3.38 eV (α‐Fe_2_O_3_/VTSC), and 2.66 eV (α‐Fe_2_O_3_/STSC), (Figure 4b). The band gaps of α‐Fe_2_O_3_/3‐APTSC and α‐Fe_2_O_3_/STSC are comparable to values reported for nanoscale hematite [19] whereas α‐Fe_2_O_3_/VTSC exhibited a higher apparent optical transition energy (3.38 eV), which may arise from ligand‐induced electronic interactions and charge‐transfer effects [20]. The observed variations in the apparent band‐gap values may be attributed to a combination of surface functionalization, nanoscale particle‐size effects, surface defects, and interfacial interactions. Further spectroscopic investigations are required to clarify the precise origin of these optical changes. Ligand‐induced shifts and new ligand to metal charge transfer (LMCT) bands are well known for TSC–metal systems, where coordination produces distinct UV–vis features and charge‐transfer bands [21]. Likewise, studies on ligand‐functionalized α‐Fe_2_O_3_ NPs have shown altered optical properties and band‐gap tuning following surface modification or composite formation [22]. Direct conjugation of TSCs to iron oxide cores has been reported to introduce additional absorption bands and to shift energies due to electronic coupling between ligand orbitals and Fe (III) centers [23]. The results indicate that the electronic and steric differences among 3‐APTSC, VTSC and STSC lead to measurable variations in band gaps and absorption maxima, demonstrating that TSC functionalization is a viable route to tailor the photophysical properties of α‐Fe_2_O_3_ NPs for catalytic, sensing, or biomedical applications.

**FIGURE 4:**
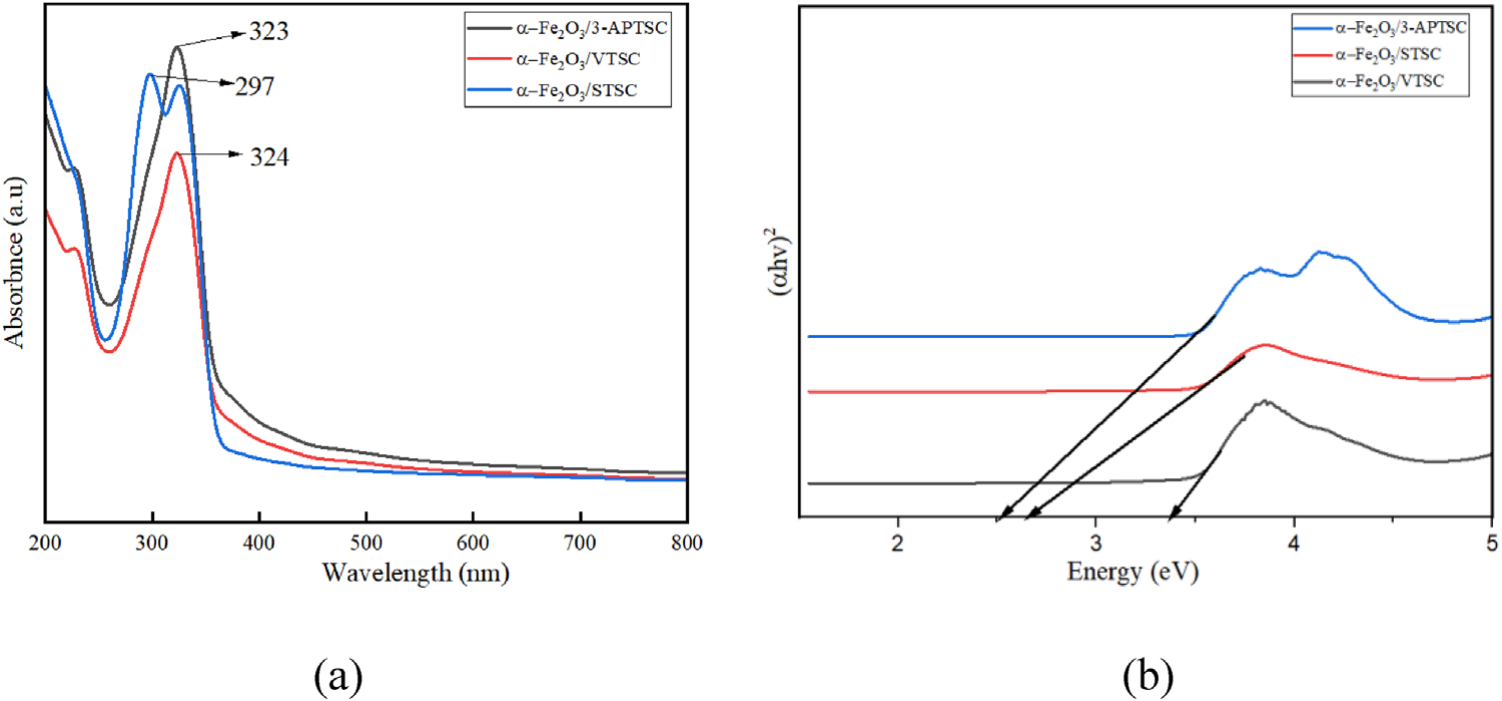
(a) UV–vis spectra and (b) band gap of α‐Fe_2_O_3_/TSC nanoconjugates.

### 4.2 FT‐IR Analysis

FT‐IR analysis provides information about the functional groups present and their binding with the NPs’ surface. The spectra of pure TSC ligands displayed characteristic peaks corresponding to ─NH, ─C═N (azomethine), ─C═S, and aromatic groups [18]. The characteristic Fe–O stretching vibration of pristine α‐Fe_2_O_3_ appeared at 574 cm^−^^1^, whereas in the nanoconjugates it was observed at 462, 460, and 453 cm^−^^1^ for α‐Fe_2_O_3_/3‐APTSC, α‐Fe_2_O_3_/VTSC, and α‐Fe_2_O_3_/STSC, respectively [24], confirming the preservation of the hematite structure after surface functionalization (Figure 5). A weak absorption around 3770 cm^−^^1^ was attributed to surface hydroxyl groups or adsorbed moisture associated with the nanoparticle surface. Absorption bands observed around 3400 cm‐^1^ together with weaker bands between 3165 and 3140 cm^−^^1^ were assigned to overlapping N–H stretching vibrations of ─NH, ─NH_2_, and azomethine‐linked N–H groups. The slight broadening and displacement of these bands suggest the involvement of hydrogen bonding and interaction with the nanoparticle surface. The azomethine (C═N) bands appeared at 1666 cm^−^^1^ (3‐APTSC), 1591 cm^−^^1^ (VTSC), and 1610 cm^−^^1^ (STSC). The thioamide‐related C═S/C─N vibrations were observed around 1130–1047 cm^−^^1^, 1282–1033 cm^−^^1^, and 1276–1060 cm^−^^1^, respectively, whereas bands at 898–891 cm^−^^1^ were assigned to N─C═S deformation [25]. These spectral changes confirm successful conjugation of the TSCs onto the surface of α‐Fe_2_O_3_ NPs, while maintaining the structural integrity of both the ligand framework and the hematite phase.

**FIGURE 5:**
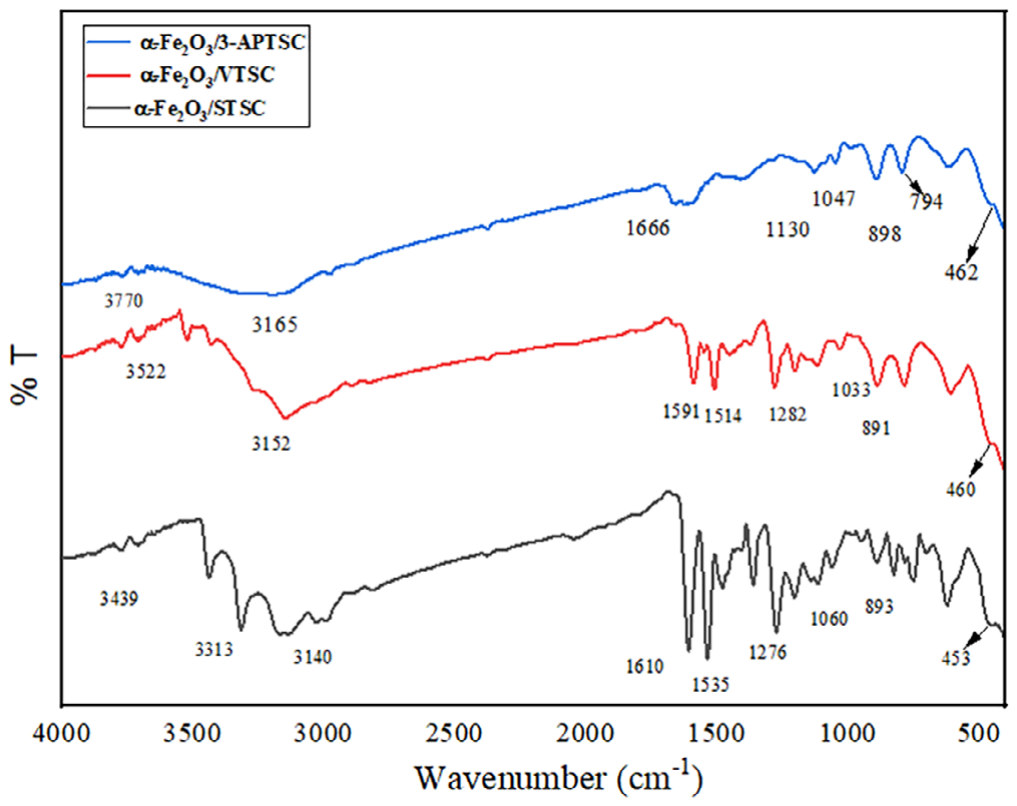
FT‐IR spectra α‐Fe_2_O_3_/TSC nanoconjugates.

### 4.3 XRD Analysis

XRD analysis was performed to investigate the crystal structure and phase composition of the synthesized nanocomposites. The diffraction patterns showed no detectable impurity peaks, confirming the high phase purity of the samples. The observed peak broadening indicated the formation of nanocrystalline materials, and the average crystallite size was calculated using the Scherrer Equation  (Equation 4).

(4) $$D=\frac{k\lambda }{\beta \cos\theta }$$

The conjugates retained the crystalline framework of hematite while showing slight peak broadening and intensity variations, which can be attributed to the presence of organic ligands on the NPs’ surface. The XRD patterns of the nanoconjugates demonstrate definitive structural evidence for successful NP formation and ligand modification. In α‐Fe_2_O_3_/STSC, prominent peaks at approximately 36.43° (2*θ*) corresponded well to characteristic planes of α‐Fe_2_O_3_ hematite (110) (JCPDS no. 33‐0664), and peaks at 21.18°, 23.51°, and 27.24° corresponds to STSC ligand and confirming the intended phase as given in Figure 6a [17, 26]. The peak broadening indicates reduced crystallite size, a phenomenon consistent with surface capping and ligand interference during crystal growth. In α‐Fe_2_O_3_/3‐APTSC, diffraction peaks were observed at around 52.94° and 60.92° (2*θ*), aligning with known iron‐oxide phases. The slightly shifted and broadened peaks at 13.08° suggested the influence of the 3‐acetylpyridine‐based TSC ligand on lattice strain and surface effects [27]. In addition, low‐angle broad peaks were attributed to the organic coating from the TSC ligand, indicating successful surface functionalization. In α‐Fe_2_O_3_/VTSC, major peaks were observed at 36.22°, 53.01°, and 60.94°. The presence of low‐angle signals between 15° and 30° further supported the incorporation of the VTSC as an amorphous layer, which may affect NPs stability and crystallite size. These observations align with recent studies on ligand‐modified α‐Fe_2_O_3_ NPs where organic moieties influence peak positions and widths [28]. To further verify the origin of the additional diffraction peaks, the XRD pattern of the pure STSC ligand was recorded (Figure 6b). The ligand exhibited several characteristic crystalline reflections in 10°–30° region. The appearance of low‐angle reflections in the α‐Fe_2_O_3_/STSC was therefore attributed to the crystalline STSC ligand incorporated onto the α‐Fe_2_O_3_ surface rather than to the formation of a new iron oxide phase. Meanwhile, the characteristic hematite reflections remain identifiable, confirming that the α‐Fe_2_O_3_ crystal structure is preserved after ligand conjugation. The absence of new intense crystalline peaks attributable to secondary iron oxide phases suggests that the hematite framework remains largely intact after conjugation [29]. The crystallite sizes determined by using the Scherrer equation were approximately 15.27, 12.47, and 11.74 nm for α‐Fe_2_O_3_/STSC, α‐Fe_2_O_3_/VTSC, and α‐Fe_2_O_3_/3‐APTSC, respectively (Tables 1, 2, 3).

**FIGURE 6:**
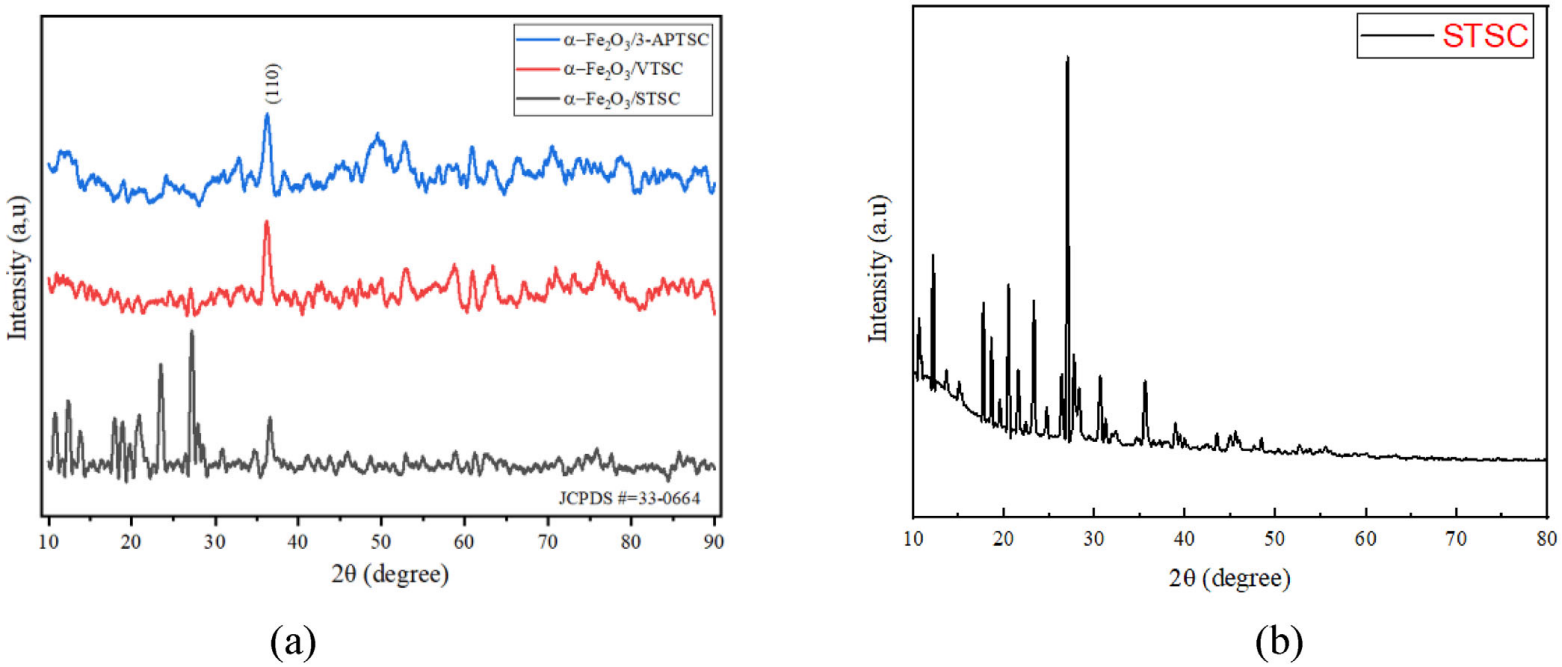
(a) XRD spectra of α‐Fe_2_O_3_/TSC nanoconjugates and (b) STSC ligand.

**TABLE 1:** XRD data of α‐Fe_2_O_3_/STSC.

S. nos.	*K*	*λ* (Å)	Peak position 2*θ* (°)	FWHM *B*_size_ (°)	*L* (nm)
1	0.94	1.54	21.18	1.06379	7.93
2	0.94	1.54	23.51	0.39703	21.34
3	0.94	1.54	27.24	0.35798	23.84
4	0.94	1.54	36.43	1.09429	7.98
Average crystalline size = 15.27 nm					

**TABLE 2:** XRD data and calculation of average size of α‐Fe_2_O_3_/VTSC.

S. nos.	*K*	*λ* (Å)	Peak position 2*θ* (°)	FWHM *B*_size_ (°)	*L* (nm)
1	0.94	1.54	15.57	1.2903	6.49
2	0.94	1.54	36.22	0.66816	13.06
3	0.94	1.54	53.01	1.20361	7.70
4	0.94	1.54	60.94	0.42523	22.63
Average crystallite size = 12.47 nm					

**TABLE 3:** XRD data and calculation of average size of α‐Fe_2_O_3_/3‐APTSC.

S. nos.	*K*	*λ* (Å)	Peak position 2*θ* (°)	FWHM *B*_size_ (°)	*L* (nm)
1	0.94	1.54	36.25	1.06225	8.22
2	0.94	1.54	52.94	1.16491	7.95
4	0.94	1.54	60.92	0.52687	18.26
Average crystallite size = 11.74 nm					

### 4.4 SEM and EDX Analysis

SEM images revealed that α‐Fe_2_O_3_ NPs exhibited nearly spherical to irregular morphologies with a noticeable tendency toward agglomeration, a typical behavior attributed to strong magnetic dipole–dipole interactions between iron oxide particles [30, 31]. The conjugation of TSC ligands may contribute to improved particle separation through surface functionalization; however, noticeable agglomeration remains present in the SEM images. Therefore, the observed morphology should be interpreted as qualitative evidence of successful surface modification rather than definitive proof of substantial agglomeration reduction (Figure 7) [32]. The EDX analyses of α‐Fe_2_O_3_/STSC, α‐Fe_2_O_3_/VTSC, and α‐Fe_2_O_3_/3‐APTSC confirmed the presence of C, O, S, and Fe in all samples, with variations reflecting the nature of each ligand. In α‐Fe_2_O_3_/STSC, C (50.09 wt%) dominated the composition, followed by O (31.04 wt%), S (12.60 wt%), and Fe (6.26 wt%). For α‐Fe_2_O_3_/VTSC, O was the major element (51.01 wt%), while Fe (29.89 wt%) formed a significant fraction, with lower contributions from carbon (14.38 wt%) and S (4.72 wt%). In α‐Fe_2_O_3_/3‐APTSC, C (41.80 wt%) and O (38.26 wt%) were the primary constituents, whereas Fe accounted for 19.10 wt% and S appeared in a minor amount (0.84 wt%) (Figure 8). These results collectively confirm successful conjugation in all three nanoconjugates, with elemental distribution influenced by the structural composition of each ligand. The EDX spectra of all the α‐Fe_2_O_3_/TSCs revealed the presence of Fe and S, though nitrogen was not detected, likely due to the limited sensitivity of EDX in capturing low atomic number elements (Z < 10), as reported in similar studies [33]. The detection of sulfur in the EDX spectra originates from the thiosemicarbazone ligands, which contain sulfur atoms within their thioamide (C═S) functional groups. Therefore, sulfur serves as an elemental indicator of successful ligand attachment to the α‐Fe_2_O_3_ NPs’ surface rather than contamination. The presence of nitrogen was generally confirmed through FT‐IR analysis, which exhibited characteristic bands for C═N stretching and N─H vibrations, indicating successful coordination through azomethine and thioamide nitrogen [34].

**FIGURE 7:**
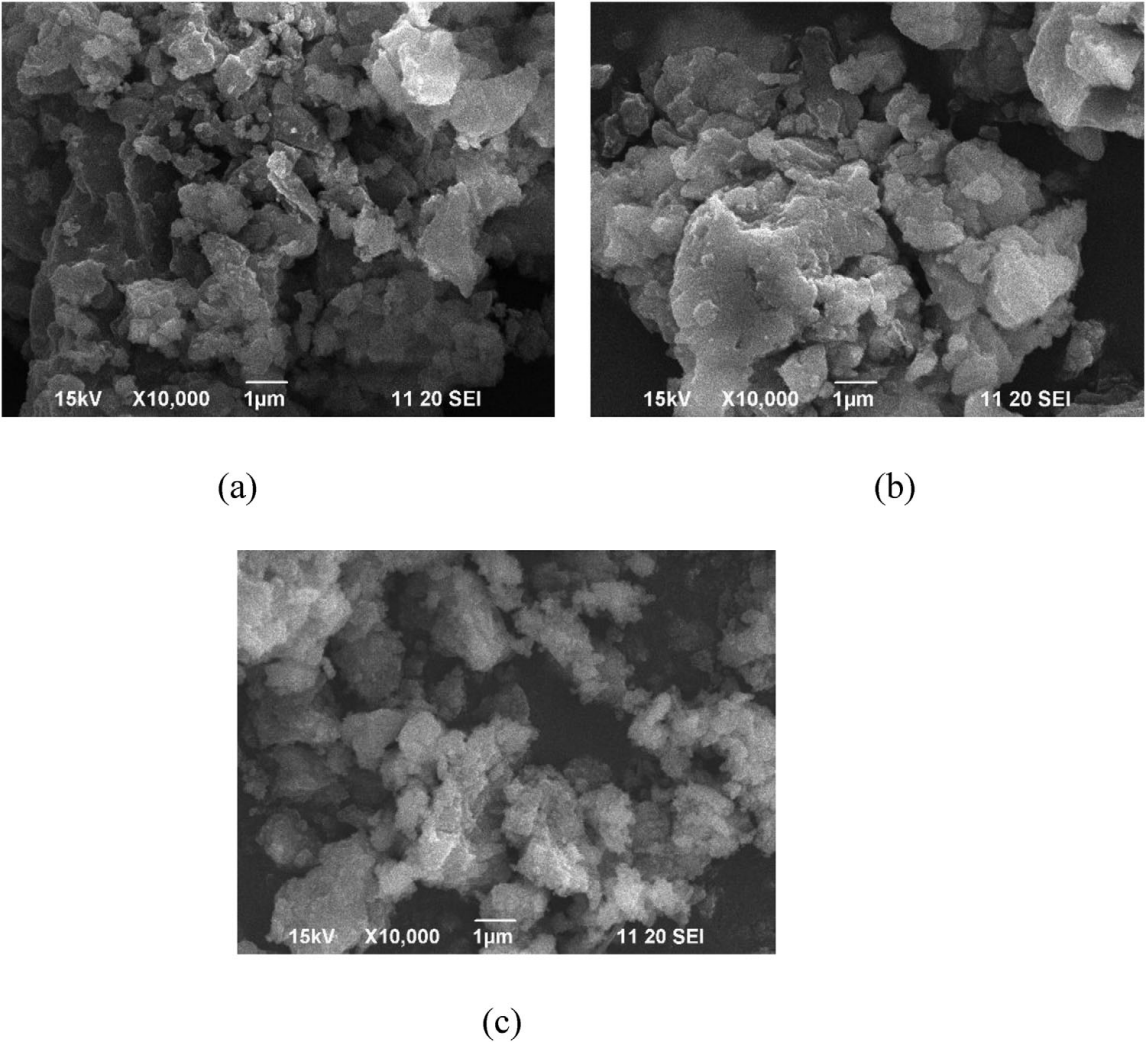
SEM images of (a) α‐Fe_2_O_3_/STSC, (b) α‐Fe_2_O_3_/VTSC, and (c) α‐Fe_2_O_3_/3‐APTSC.

**FIGURE 8:**
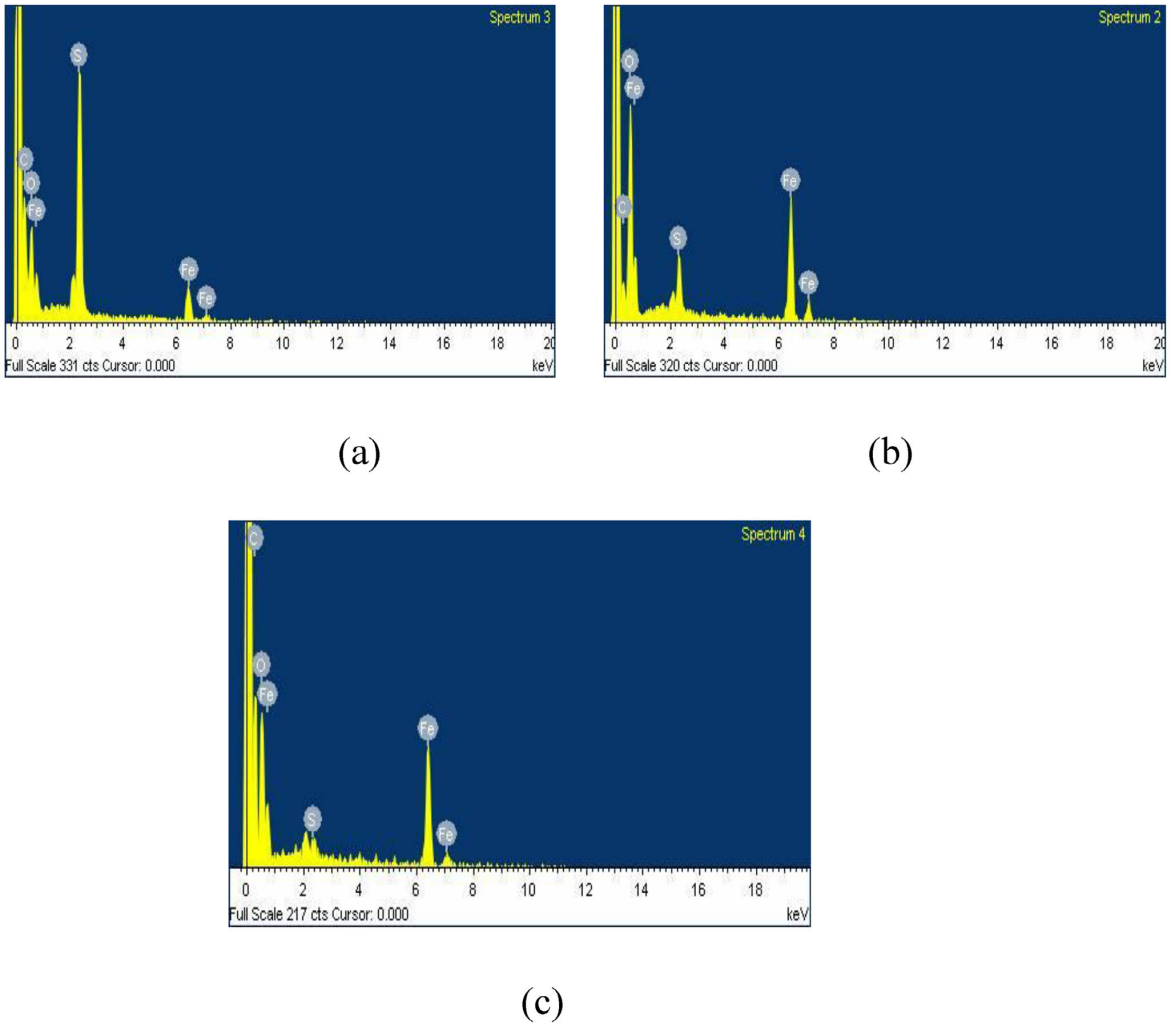
EDX of (a) α‐Fe_2_O_3_/STSC, (b) α‐Fe_2_O_3_/VTSC, and (c) α‐Fe_2_O_3_/3‐APTSC.

### 4.5 Zeta Potential Analysis

Zeta potential measurements showed negative surface charges of –7.06, –13.7, and –13.9 mV for α‐Fe_2_O_3_/VTSC, α‐Fe_2_O_3_/STSC, and α‐Fe_2_O_3_/3‐APTSC, respectively (Figure 9). Although these values are lower than the ±30 mV generally associated with highly electrostatically stable colloids, the increased negative surface charge observed for α‐Fe_2_O_3_/STSC and α‐Fe_2_O_3_/3‐APTSC suggests improved dispersion compared with α‐Fe_2_O_3_/VTSC. The enhanced stability may be attributed to the surface‐bound TSC ligands, which provide additional steric stabilization alongside moderate electrostatic repulsion [35]. These findings suggest that conjugation with thiosemicarbazone derivatives improves the dispersion characteristics and colloidal stability of α‐Fe_2_O_3_ NPs, with α‐Fe_2_O_3_/3‐APTSC showing the most favorable colloidal properties. Similar behavior has been reported for ligand‐functionalized iron oxide NPs [36].

**FIGURE 9:**
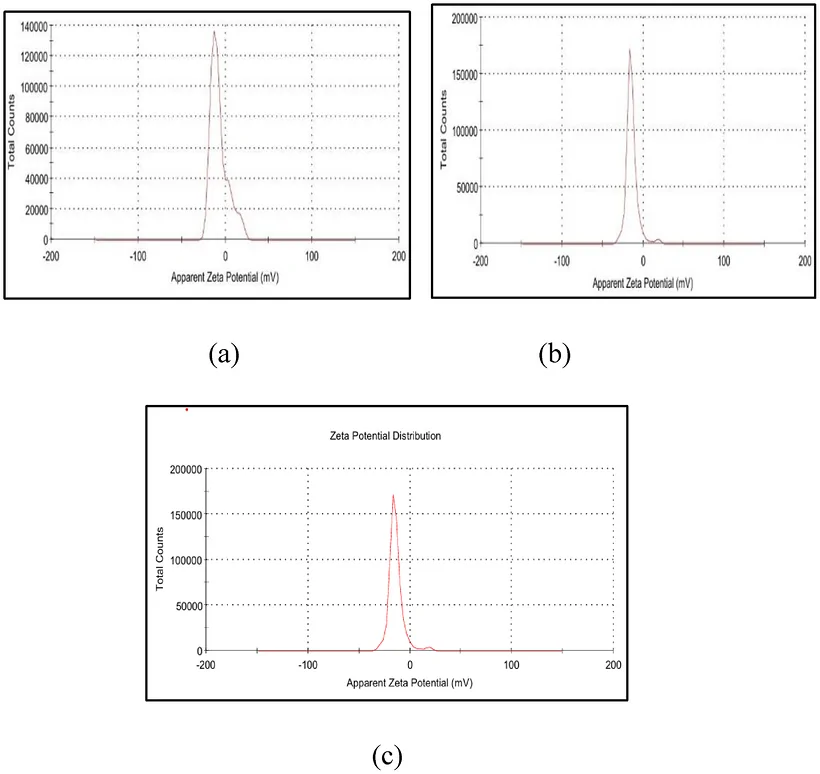
Zeta potential distribution curves of (a) α‐Fe_2_O_3_/VTSC, (b) α‐Fe_2_O_3_/STSC, and (c) α‐Fe_2_O_3_/3‐APTSC.

### 4.6 Antibacterial Activity Evaluation

The antibacterial efficacy of α‐Fe_2_O_3_/TSCs was evaluated at 50, 75, and 100 µg/mL (Table 4). The results demonstrated a concentration‐dependent enhancement in the antibacterial activity, with inhibition zones increasing at higher NPs concentrations. α‐Fe_2_O_3_/3‐APTSC exhibited the most significant antibacterial activity across multiple strains. It displayed substantial inhibition against *S. abony* (17.33 ± 0.58 mm) and *S. aureus* (16.67 ± 0.58 mm) and the highest inhibition zones against *E. coli* (18.00 ± 0.00 mm) and *B. subtilis* (17.33 ± 0.58 mm) at 100 µg/mL, which are somewhat comparable to the standard antibiotic gentamicin (20.33–21.33 mm), whereas the other nanocomposites showed negligible or no activity toward these pathogens. In contrast, α‐Fe_2_O_3_/STSC was inactive against *S. abony* and *S. aureus* but showed moderate antibacterial activity, particularly against *E. coli* (18.00 ± 0.58 mm) and *B. subtilis* (18.33 ± 0.58 mm). α‐Fe_2_O_3_/VTSC exhibited lower activity, with inhibition zones below 15 mm. The agar plates of α‐Fe_2_O_3_/TSCs are shown in Figure 10. Overall, α‐Fe_2_O_3_/3‐APTSC exhibited broad‐spectrum and superior antibacterial activity, likely due to the enhanced surface functionalization and electron‐donating nature of the amino group in the 3‐APTSC ligand, which improves the interaction with bacterial cell membranes and facilitates ROS generation. The observed trends indicate that ligand substitution significantly influences antibacterial efficacy, with 3‐APTSC providing a synergistic enhancement to the intrinsic activity of α‐Fe_2_O_3_ NPs. Their antimicrobial effects are attributed primarily to ROS generation, cell‐membrane disruption and metal ion release [37]. For example, a well‐diffusion study of Fe_3_O_4_ NPs coated with PEG and loaded with gentamicin showed inhibition‐zone diameters of up to ∼26 mm against *A. baumannii*, *P. mirabilis*, and *S. epidermidis* at 100 µg/mL, with MIC values as low as 25 µg/mL [38]. The size, surface charge, doping and functionalization significantly influence antibacterial potency [39]. These findings underscore that surface‐modified iron oxide nanocomposites hold potential as next‐generation antibacterial agents, especially in combating antibiotic‐resistant pathogens. Fe_3_O_4_@SiO_2_ core–shell NPs were synthesized using coprecipitation and microwave‐assisted hydrothermal methods and subsequently loaded with three essential oils: thyme, rosemary, and basil, to improve their stability and bioavailability. In vitro antimicrobial studies demonstrated that the essential‐oil‐loaded nanocomposites effectively inhibited the growth of *S. aureus, P. aeruginosa, E. coli*, and *C. albicans*, indicating their potential as efficient platforms for antimicrobial therapies [40]. Compared with bare α‐Fe_2_O_3_ NPs, α‐Fe_2_O_3_/3‐APTSC demonstrated broader antibacterial activity with appreciable inhibition against *S. abony* at 100 µg/mL [17]. This enhanced spectrum of activity suggests that conjugation with the 3‐APTSC ligand improves the antibacterial efficacy of α‐FeO_3_ NPs.

**TABLE 4:** Antibacterial activity of α‐Fe_2_O_3_/TSC nanoconjugates.

S. nos.	Bacterial strain	Nanoconjugate	Concentration (µg/mL)			Gentamicin (standard) (mm)
			50	75	100	50 µg/mL
			Zone of inhibition(mm)			
1.	*B. subtilis*	α‐Fe_2_O_3_/STSC	15.33±0.58	17.67±1.15	18.33±0.58	20.33±0.58
		α‐Fe_2_O_3_/VTSC	0.00±0.00	0.00±0.00	0.00±0.00	21.25±0.41
		α‐Fe_2_O_3_/3‐APTSC	13.00±0.58	14.33±1.26	17.33±0.58	20.67±0.58
2.	*S. aureus*	α‐Fe_2_O_3_/STSC	0.00±0.00	0.00±0.00	0.00±0.00	26.7±0.58
		α‐Fe_2_O_3_/VTSC	0.00±0.00	0.00±0.00	0.00±0.00	22.41±0.53
		α‐Fe_2_O_3_/3‐APTSC	12.67±0.58	14.33±0.58	16.67±0.58	26.7±0.58
3.	*E. coli*	α‐Fe_2_O_3_/STSC	15.67±1.15	17.67±1.15	18.00±0.58	20.33±0.58
		α‐Fe_2_O_3_/VTSC	12.33±0.58	13.67±0.58	15.00±0.00	22.33±0.58
		α‐Fe_2_O_3_/3‐APTSC	13.67±0.58	15.67±0.8	18.00±0.00	21.33±0.58
4.	*S. abony*	α‐Fe_2_O_3_/STSC	0.00±0.00	0.00±0.00	0.00±0.00	26.7±0.58
		α‐Fe_2_O_3_/VTSC	0.00±0.00	0.00±0.00	0.00±0.00	20.67±0.58
		α‐Fe_2_O_3_/3‐APTSC	12.33±0.58	14.00±0.58	17.33±0.58	20.67±0.58

**FIGURE 10:**
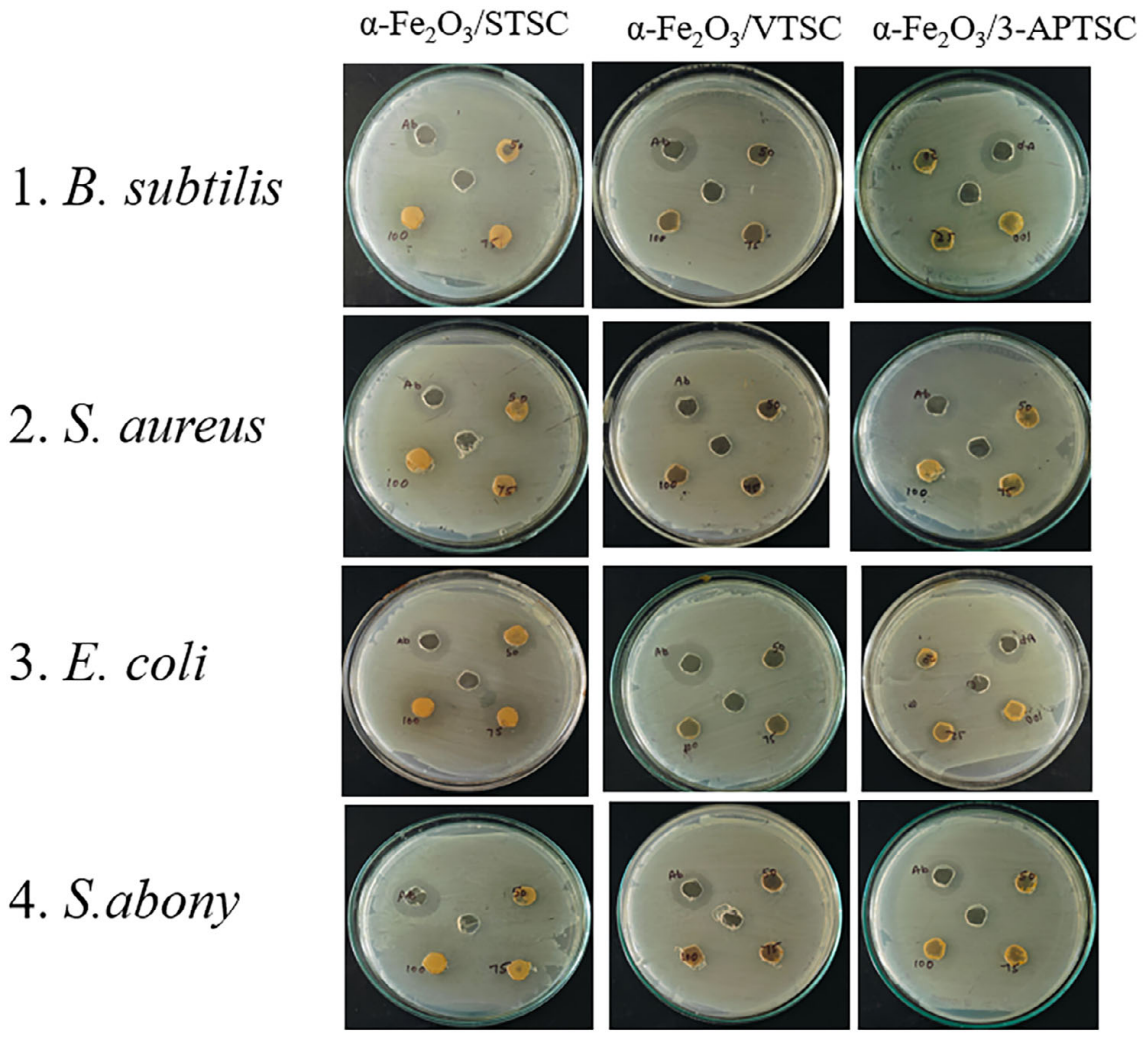
Agar plates showing antibacterial activity of α‐Fe_2_O_3_/STSC, α‐Fe_2_O_3_/VTSC, and α‐Fe_2_O_3_/3‐APTSC.

### 4.7 Antioxidant Activity

α‐Fe_2_O_3_/3‐APTSC exhibited the highest scavenging percentage activity of 74.47%, followed by α‐Fe_2_O_3_/STSC with 63.97% and α‐Fe_2_O_3_/VTSC 42.96% as given in Table 5. As a reference standard, ascorbic acid displayed an SPA of 82.35% under the same conditions. These results indicate that α‐Fe_2_O_3_/3‐APTSC showed the highest antioxidant activity among the nanoconjugates. The DPPH scavenging activities of α‐Fe_2_O_3_/STSC and α‐Fe_2_O_3_/3‐APTSC were higher than those of α‐Fe_2_O_3_/VTSC. The IC_50_ values of DPPH radical scavenging activities of α‐Fe_2_O_3_/STSC and α‐Fe_2_O_3_/3‐APTSC showed 50% inhibition at 40 µg/mL, respectively. However, no significant differences in DPPH scavenging potential were found among the α‐Fe_2_O_3_/TSCs at 75 µg/mL. The lower IC_50_ value of the nanoconjugates indicates a stronger DPPH scavenging ability. The triplicate values were expressed as mean ± standard deviation and the antioxidant activity results are presented in Table 5. STSC and 3‐APTSC showed the highest (p ≤ 0.05) scavenging activity. Iron‐oxide nanocomposites especially those prepared by green routes or surface‐functionalized with phenolic ligands, have shown significant antioxidant activity in vitro as shown previously. Gallic‐acid‐functionalized Fe‐oxide NPs (GA‐IONPs) reduced intracellular ROS by ≈70% at 0.5 mg·mL^−^^1^ and protected human dermal papilla cells from H_2_O_2_‐induced oxidative stress [41]. Green‐synthesized Fe_x_O_y_ NPs prepared with plant polyphenols also showed concentration‐dependent DPPH and ABTS radical scavenging, often matching or exceeding activities of chemically synthesized counterparts [42]. Surface functionalization with natural antioxidants (e.g., gallic acid) improves dispersion and enhances radical‐scavenging and reducing power in common assays [43]. Various studies have shown that the functionalized iron oxides undergo different phytochemical capping or hybridization like with graphene oxide or digitonin imparts improved antioxidant capacity and biocompatibility, making such nanocomposites promising for biomedical and protective applications [44, 45].

**TABLE 5:** Antioxidant activity of α‐Fe_2_O_3_/TSC nanoconjugates.

S. nos.	Samples	DPPH (absorbance at 517 nm)	SPA (%)	IC_50_ value (µg/mL)
1	Control	0.952 (±0.021)		
2	Ascorbic acid	0.168±.002	82.35	10.26±0.68
3	α‐Fe_2_O_3_/STSC	0.343±.026	63.97	45.26±0.16
4	α‐Fe_2_O_3_/VTSC	0.543±.020	42.96	80.05±0.52
5	α‐Fe_2_O_3_/3‐APTSC	0.243±.033	74.47	40.55±0.09

The same research group has previously reported the antioxidant activity of ZnO/TSC nanoconjugates, in which ZnO/3‐APTSC demonstrated superior activity as compared to the other counterparts [46], whereas in another series, CuO/VTSC exhibited highest scavenging activity [18]. However, α‐Fe_2_O_3_/STSC, and α‐Fe_2_O_3_/3‐APTSC have shown better antioxidant potential compared to the CuO and ZnO counterparts, as shown in Figure 11. Overall, conjugation with 3‐APTSC generally resulted in improved antioxidant activity for iron and zinc oxide systems, likely due to favorable electron‐donating interactions. These results indicate that the antioxidant behavior of TSC‐based nanoconjugates is strongly influenced by both the nature of the metal oxide and the specific TSC ligand.

**FIGURE 11:**
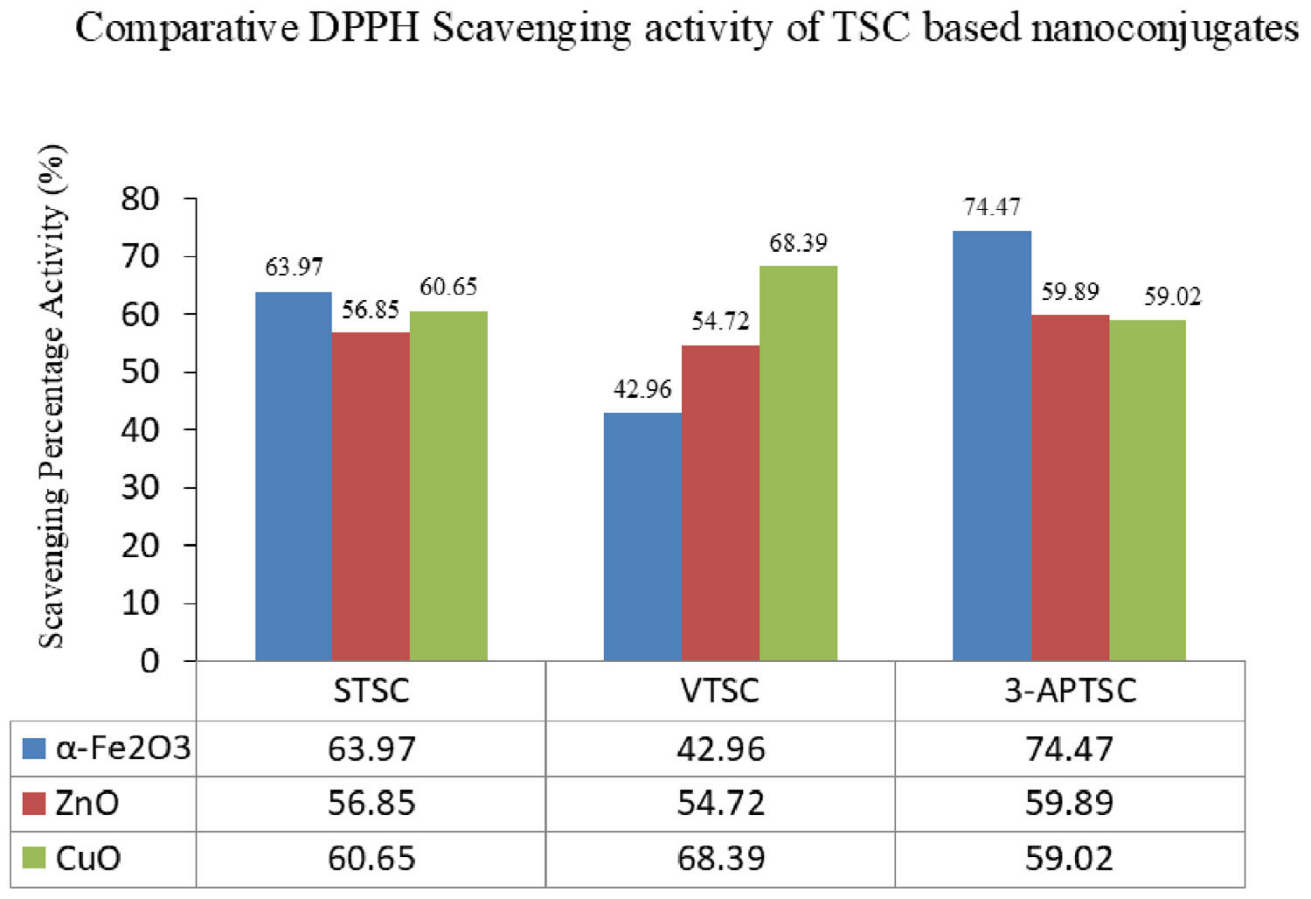
Comparative DPPH radical scavenging activity (%) of STSC, VTSC, and 3‐APTSC‐conjugated nanomaterials based on α‐Fe_2_O_3_, ZnO, and CuO NPs. Each value is the mean of three replicates, and vertical bars indicate the standard error.

### 4.8 Photodegradation of MG Dye

Nanocomposites have been playing a very significant role as photocatalysts in wastewater remediation, enabling the degradation of persistent pollutants into less harmful products, due to their high surface area and favorable charge‐transfer properties, enhancing light absorption and suppressing electron–hole recombination, leading to improved catalytic efficiency [46]. Ferrite NPs, particularly CuFe_2_O_4_, have attracted attention due to their visible‐light activity, photo‐Fenton behavior, and magnetic recoverability, which allows easy separation and reuse [47]. Recently, rGO‐based metal oxide nanocomposites have demonstrated superior performance by promoting rapid charge transport and providing abundant active sites, resulting in enhanced photocatalytic and antimicrobial activities. Notably, a B–CuO/rGO nanocomposite achieved 98% degradation of methylene blue within 90 min, highlighting the potential of such hybrid systems for sustainable wastewater treatment [48]. In another study, the α‐Fe_2_O_3_/rGO composite exhibited enhanced visible‐light photocatalytic activity due to improved charge carrier transfer and increased magnetic response compared to pure α‐Fe_2_O_3_ NPs. The composite with an α‐Fe_2_O_3_:rGO mass ratio of 10:1 showed the highest performance, achieving 60% degradation of Congo red within 30 min under visible‐light irradiation [49]. α‐Fe_2_O_3_, a visible‐light‐responsive semiconductor with a band gap of ∼2.1 eV, is widely used in photocatalysis but often suffers from charge recombination and surface instability. Previously, α‐Fe_2_O_3_/rGO composite was synthesized to detect rhodamine 6G (R6G), showing high photocatalytic activity, even after 10 cycles [50]. In another study, the phytoextract‐assisted synthesis of α‐Fe_2_O_3_/MgO nanocomposite and its photocatalytic efficiency were tested against GV under visible light. Under optimized conditions (100 mg/L catalyst dose, 3 mg/L initial dye, pH 10), the composite reached 96.2% degradation of GV, whereas bare α‐Fe_2_O_3_ achieved 80.2%. This highlights that supporting hematite with MgO and using plant‐extract‐assisted synthesis can substantially elevate photocatalytic performance [51]. In this study, the photocatalytic degradation of MG dye under visible light irradiation was systematically evaluated using α‐Fe_2_O_3_ conjugated with various TSC ligands.

In the present study, the degradation of MG was monitored spectrophotometrically at 617 nm, and the data were analyzed using a pseudo‐first‐order kinetic model. Among the conjugates, α‐Fe_2_O_3_/STSC demonstrated a degradation efficiency of 65% after a fixed exposure period (Figure 12a). The corresponding pseudo‐first‐order rate constant (*k*), derived from the linear fit of ln(*C*_0_/*C*) versus time, was calculated to be 0.01231 min^−^^1^ with a high correlation coefficient (*R* ^2^ = 0.9767), confirming a satisfactory kinetic fit (Figure 12b). α‐Fe_2_O_3_/VTSC showed a dye degradation efficiency of 63% under similar conditions (Figure 13a). Kinetic analysis yielded a rate constant of 0.01449 min^−^^1^ with *R* ^2^ = 0.9651, indicating a slightly faster reaction rate compared to STSC despite a marginally lower overall degradation efficiency (Figure 13b). The highest degradation efficiency of 68% was achieved by α‐Fe_2_O_3_/3‐APTSC (Figure 14a). However, the corresponding rate constant (k = 0.01275 min^−^^1^; *R* ^2^ = 0.9440) was modestly lower than that of VTSC, suggesting that factors other than reaction kinetics may influence overall degradative performance (Figure 14b).

**FIGURE 12:**
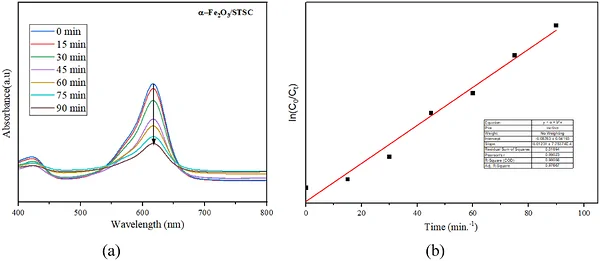
(a) UV–vis spectra exhibiting photodegradation of MG dye, and (b) pseudo‐first‐order kinetics of α‐Fe_2_O_3_/STSC.

**FIGURE 13:**
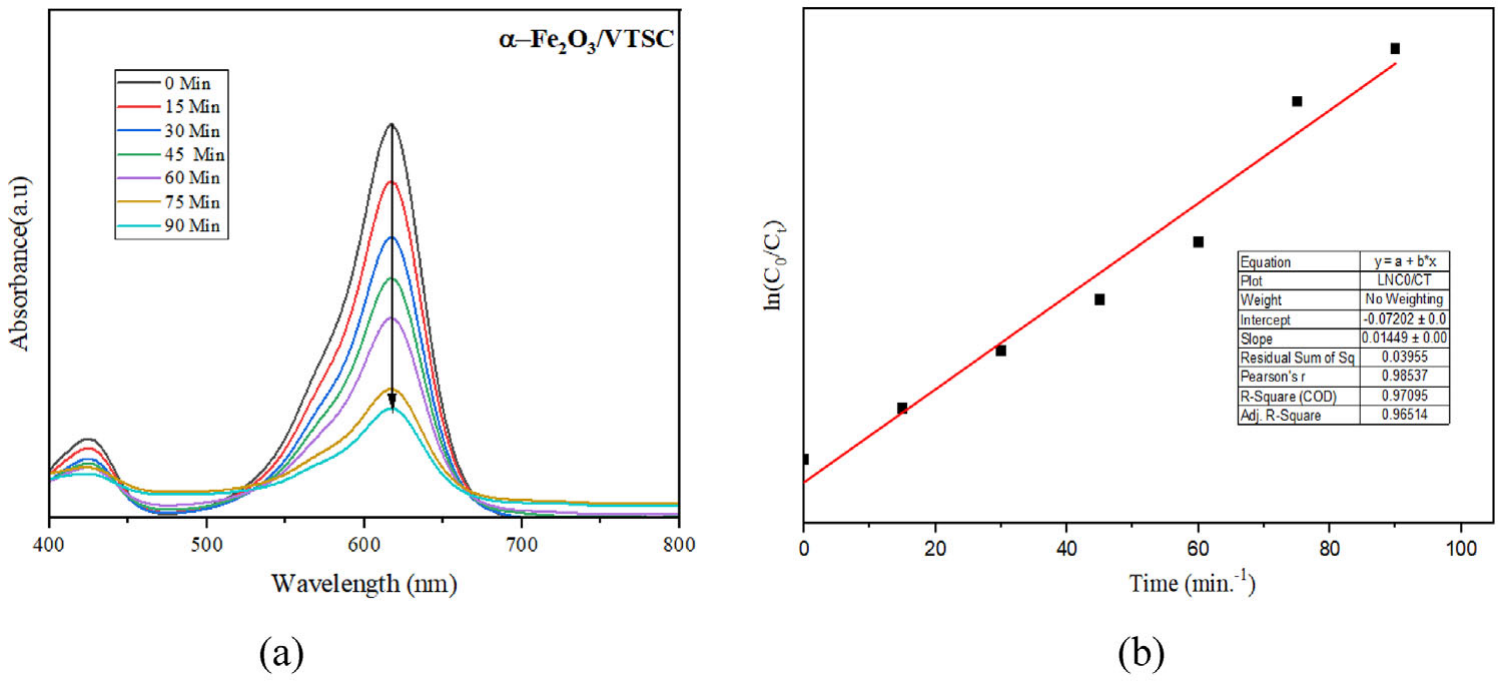
(a) UV–vis spectra exhibiting photodegradation of MG dye, and (b) pseudo‐first‐order kinetics of α‐Fe_2_O_3_/VTSC.

**FIGURE 14:**
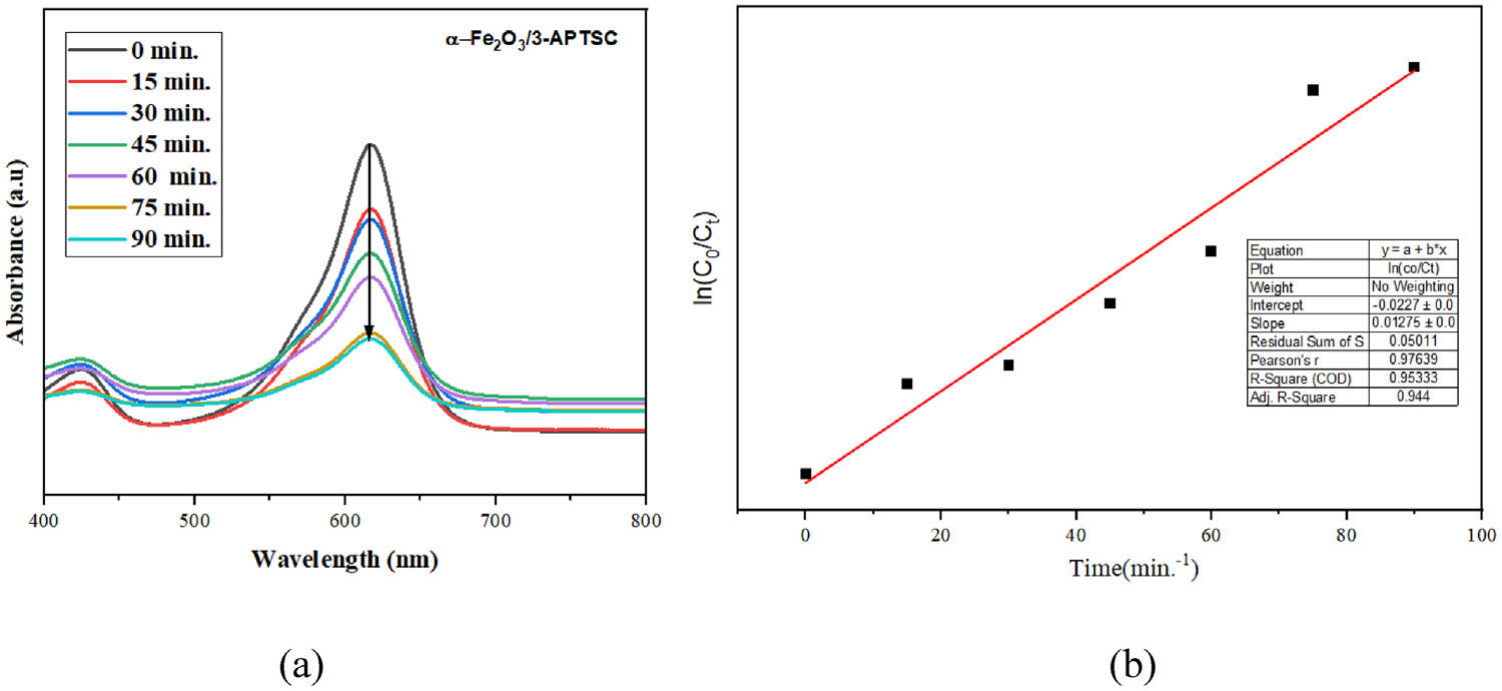
(a) UV–vis spectra exhibiting photodegradation of MG dye, and (b) pseudo‐first‐order kinetics of α‐Fe_2_O_3_/3‐APTSC.

The enhanced photocatalytic performance of the α‐Fe_2_O_3_/TSC nanoconjugates compared to bare α‐Fe_2_O_3_ NPs demonstrates the beneficial effect of TSC functionalization. Among the synthesized nanoconjugates, α‐Fe_2_O_3_/3‐APTSC achieved the highest dye degradation efficiency, whereas α‐Fe_2_O_3_/VTSC exhibited the highest degradation rate constant. The improved photocatalytic activity can be attributed to enhanced dye adsorption and more efficient charge separation promoted by the TSC ligands. Furthermore, photoexcited electrons and holes generate reactive oxygen species, including superoxide (O_2_•^−^) and hydroxyl (•OH) radicals, which oxidatively degrade malachite green into smaller, less harmful products [52, 53]. Some of the IONPs nanocomposites and their photocatalytic activity are given in Table 6.

**TABLE 6:** Some previously reported IONPs and their photocatalytic activity.

S. nos.	Photocatalysts	Dye(s)	Light source	Degradation (%)	Time (min)	References
1	Fe_3_O_4_ NPs	BG and RhB	Visible light	80.4 and 36	180	[54]
2	α‐Fe_2_O_3_/Fe_3_O_4_/g‐C_3_N_4_	RhB	Visible light	58	60	[55]
3	α‐Fe_2_O_3_ NPs	MB	Sun light	33	120	[56]
4	Fe_2_O_3_/Cu_2_O	RhB, MB, and CV	Visible light	85	90–120	[57]
5	γ‐Fe_2_O_3_/TiO_2_	Orange G	White LED	95	60	[58]
**6**	α‐Fe_2_O_3_/STSC, α‐Fe_2_O_3_/VTSC, and α‐Fe_2_O_3_/3‐APTSC	MG	Visible light	65, 63, and 68	90	Present work

Abbreviations: BG, brilliant green; RhB = rhodamine B; MB = methylene blue; MO = methyl orange; CV = crystal violet; MG = malachite green.

## 5 Conclusion

In this study, α‐Fe_2_O_3_ NPs synthesized through a green route were successfully functionalized with three thiosemicarbazone ligands (STSC, VTSC, and 3‐APTSC) using a hydrothermal conjugation method. Structural characterization by UV–vis spectroscopy, FT‐IR, XRD, and SEM–EDX confirmed the formation of α‐Fe_2_O_3_/TSC nanoconjugates and successful surface functionalization. XRD analysis revealed nanoscale crystallite sizes of the conjugates.

The biological and photocatalytic evaluations demonstrated that conjugation with TSCs enhanced the functional performance of α‐Fe_2_O_3_ NPs. Among the synthesized conjugates, α‐Fe_2_O_3_/3‐APTSC was the most versatile, showing the highest DPPH radical scavenging activity (74.47%), broad‐spectrum antibacterial activity with inhibition zones up to 18.00 mm, and the highest photocatalytic degradation efficiency (68%) toward malachite green dye under visible‐light irradiation. The photocatalytic degradation followed pseudo‐first‐order kinetics with rate constants ranging from 0.01231 to 0.01449 min^−^^1^. These findings indicate that the nature of the ligand used for functionalization influences the physicochemical and functional properties of the resulting nanoconjugates. However, detailed colloidal stability studies, including dynamic light scattering, should be undertaken in future to fully elucidate the electronic interactions between the NPs’ surface and the coordinated TSCs. Furthermore, photocatalytic experiments were conducted under a single set of reaction conditions, and comprehensive optimization studies involving catalyst dosage, dye concentration, pH, and irradiation parameters were beyond the scope of this investigation. Overall, the results demonstrate that α‐Fe_2_O_3_/TSC nanoconjugates represent promising multifunctional materials with potential applications in environmental remediation and antimicrobial therapies. Evaluation of cytotoxicity and biocompatibility to explore their broader biomedical and environmental applicability should also be explored.

## Author Contributions

**Ekhlakh Veg**: investigation, formal analysis, interpretation, and writing – original and final draft. **Srishti Dwivedi**: characterization and proof‐reading, **Smita Rai**: antibacterial and antioxidant activity evaluation. **Swati Sharma**: antibacterial and antioxidant activity evaluation. **Nashra Fatima**: photocatalytic activity. **Seema Joshi**: co‐supervision and proofreading. **Abdul Rahman Khan**: resources. **Tahmeena Khan**: conceptualization, supervision, and writing – final draft and proof‐reading.

## Funding

The authors have nothing to report.

## Ethics Statement

The authors have nothing to report.

## Conflicts of Interest

The authors declare no conflicts of interest.

## Data Availability

Data sharing not applicable to this article as no datasets were generated or analyzed during the current study.
